# Fructose malabsorption and fructan malabsorption are associated in patients with irritable bowel syndrome

**DOI:** 10.1186/s12876-024-03230-x

**Published:** 2024-04-24

**Authors:** Twan Sia, Riki O. Tanaka, Albert Mousad, Aditya P. Narayan, Kristen Si, Leeon Bacchus, Hind Ouerghi, Aashka Patel, Arnav Patel, Evan Cunningham, Taylor Epstein, Jerry Fu, Stanley Liu, Raisa Khuda, Paige McDonald, Shibani Mallik, Joanna McNulty, Michelle Pan, John Leung

**Affiliations:** 1 Boston Specialists, 65 Harrison Ave #201, Boston, MA 02111 USA; 2 Stanford University School of Medicine, 291 Campus Drive, Stanford, CA 94305 USA; 3 Tufts University School of Medicine, 145 Harrison Ave, Boston, MA 02111 USA

**Keywords:** IBS, Inulin, Food intolerance, Hydrogen breath test, FODMAP, Disorders of gut-brain interaction, DGBI

## Abstract

**Background:**

Food malabsorption and intolerance is implicated in gastrointestinal symptoms among patients with irritable bowel syndrome (IBS). Key triggers include fructose and fructan. Prior studies examined fructose and fructan malabsorption separately in IBS patients. None have concurrently assessed both within the same patient group. We aimed to investigate the association between fructose and fructan malabsorption in the same patients with IBS using hydrogen breath testing (HBT).

**Methods:**

We retrospectively identified patients with IBS who underwent fructose and fructan HBTs and abstracted their results from the electronic medical record. Fructose and fructan HBTs were performed by administering a 25 g fructose solution or 10 g fructan solution, followed by breath hydrogen readings every 30 min for 3 h. Patients were positive for fructose or fructan malabsorption if breath hydrogen levels exceeded 20 ppm.

**Results:**

Of 186 IBS patients, 71 (38.2%) were positive for fructose malabsorption and 91 (48.9%) were positive for fructan malabsorption. Of these patients, 42 (22.6%) were positive for fructose malabsorption and fructan malabsorption. Positive fructose HBT readings were significantly associated with positive fructan HBT readings (*p* = 0.0283). Patients positive for fructose malabsorption or fructan malabsorption had 1.951 times higher odds of testing positive for the other carbohydrate.

**Conclusions:**

Our results reveal a clinically significant association between fructose malabsorption and fructan malabsorption in patients with IBS. Fructan malabsorption should be assessed in patients with fructose malabsorption, and vice versa. Further studies are required to identify the mechanisms underlying our findings.

**Supplementary Information:**

The online version contains supplementary material available at 10.1186/s12876-024-03230-x.

## Introduction

Irritable bowel syndrome (IBS) is a common chronic functional bowel disorder characterized by abdominal pain, distension, and changes in the frequency or appearance of stool [1, 2]. Various factors contribute to the pathophysiology of IBS, including food sensitivities [1, 3]. Over 80% of patients with IBS attribute their symptoms to diet or specific foods [4].

FODMAPs (Fermentable Oligo-, Di-, Monosaccharides, and Polyols) are carbohydrates that are malabsorbed in the small intestine. Therefore, FODMAPs reach the distal ileum and colon where they undergo fermentation by gut microbiota to produce short-chain fatty acids and gases such as hydrogen, thus distending the lumen and triggering symptoms [1, 5]. FODMAPs also generate an osmotic force that draws water into the large intestines, resulting in diarrhea and/or bloating [3, 6]. The low FODMAP diet has been shown to improve symptoms in patients with IBS [7–10]; however, the restrictiveness of the low FODMAP diet can be prohibitive for long term patient adherence [11–13]. Thus, identifying specific food triggers within the low FODMAP diet has remained a critical area of research in IBS management [14, 15].

Fructose and fructans are FODMAPs that have been of interest due to their prevalence in modern diets. Fructose is a dietary monosaccharide commonly found in fruits, vegetables, honey, and artificial sweeteners such as high fructose corn syrup [1]. In contrast, fructan is a polysaccharide composed of multiple fructose units with a terminal glucose unit commonly contained in wheat-based products (cereals, wheat, rye), vegetables (onions, shallots, leeks, asparagus, artichokes, beets, brussels sprouts), and fruits (watermelon, grapefruit, nectarine, persimmon, plums, pomegranate, ripe bananas). Fructose malabsorption is proposed to be the result of defective fructose transporter proteins with resultant excess fructose in the distal ileum which undergo bacterial fermentation [16]. On the other hand, the human small intestine epithelium lacks the enzymes necessary for hydrolyzing the glycosidic linkages in fructan. Thus, a majority of fructan sugars pass through to the large intestine, where they osmotically draw in water into the colonic lumen and undergo bacterial fermentation to produce gas. Due to visceral hypersensitivity in patients with IBS, fructan malabsorption may therefore result in gastrointestinal symptoms [17].

While there are no standardized diagnostic tests for fructose or fructan malabsorption, the hydrogen breath test (HBT) is the most accepted and well-studied [18]. Fructose and fructan HBTs are executed similarly to HBTs used to diagnose small intestinal bacterial overgrowth (SIBO) and lactose intolerance, the most widely accepted indications for HBT [19], but with ingestion of different carbohydrate solutions. Previous primary literature on breath tests for identifying fructose malabsorption or fructan malabsorption is summarized in Table 1 [20–44]. Fructose HBTs have been relatively more well-studied than fructan HBTs, which have been primarily limited to preliminary reports. Of note, there is heterogeneity in previous literature for fructose and fructan HBTs in terms of carbohydrate solution dose.

**Table 1:** Review of primary literature on fructose breath tests and fructan breath tests

Substrate	Gas Reading	Diagnostic Criteria for Malabsorption	Proportion of Malabsorption	Reference
Fructose breath tests				
Fructose (A. 15 g fructose in 150 cc water; B. 25 g fructose in 250 cc water; C. 50 g fructose in 500 cc water; D. 50 g fructose in 125 cc water)	Hydrogen and methane	Rise of ≥ 20 ppm over baseline hydrogen, methane, or both; or successive rise of ≥ 5 ppm over baseline in 3 consecutive readings. Test duration of 4–6 h	0% (A), 10% (B), 80% (C), and 60% (D) in healthy patients (n = 20 each)	Rao et al., 2007 [20]
Fructose (50 g fructose)	Hydrogen and methane	Hydrogen levels ≥ 20 ppm; methane levels ≥ 12 ppm; or sum of combined peaks ≥ 15 ppm. Test duration of 2–4 h	55% in IBS patients (n = 94), and 65% in IBS patients on fructose-reduced diet (n = 88)	Berg et al., 2013 [21]
Fructose (25 g fructose in 200 cc water)	Hydrogen	Rise of ≥ 20 ppm over baseline hydrogen. Test duration of 2 h	16% in lactose intolerant patients (n = 279)	Schnedl et al., 2020 [22]
Fructose (1 g/kg fructose up to maximum of 25 g)	Hydrogen and methane	Rise of ≥ 20 ppm over baseline hydrogen. Test duration of 2.5 h	55% in patients with abdominal symptoms (n = 222)	Escobar et al., 2014 [23]
Fructose (35 g in 300 cc water)	Hydrogen and methane	Rise of ≥ 20 ppm over baseline hydrogen; or ≥ 10 ppm over baseline methane twice in succession. Test duration of 5 h	45% in functional gastrointestinal disease patients (n = 1372)	Wilder-Smith et al., 2013 [24]
Fructose (1 g/kg fructose up to maximum of 25 g)	Hydrogen	Rise of ≥ 20 ppm over baseline hydrogen. Test duration of 3 h	35% in pediatric patients with gastrointestinal symptoms (n = 323)	Kwiecień et al., 2021 [25]
Fructose (35 g fructose in 300 cc water)	Hydrogen and methane	Rise of ≥ 20 ppm over baseline hydrogen; or ≥ 10 ppm over baseline methane. Test duration of 5 h	27% in functional gastrointestinal disease patients (n = 30)	Wilder-Smith et al., 2021 [26]
Fructose (A. 50 g fructose, B. 25 g fructose)	Hydrogen	Rise of ≥ 20 ppm over baseline hydrogen. Test duration of 2 h	58% (A) and 19% (B) in healthy patients (n = 21)	Truswell et al., 1988 [27]
Fructose (1 g/kg fructose up to maximum of 25 g)	Hydrogen	Rise of ≥ 20 ppm over baseline hydrogen. Test duration of 2 h	30% in pediatric patients with chronic abdominal pain (n = 118)	Posovszky et al., 2019 [28]
Fructose (1 g/kg fructose up to maximum of 25 g)	Hydrogen and methane	Rise of ≥ 20 ppm over baseline hydrogen; rise of ≥ 5 to ≥ 12 ppm methane; or ≥ 10 to ≥ 15 ppm for hydrogen-plus-methane. Test duration of 3 h	50% in pediatric patient with abdominal symptoms (n = 54)	Hammer et al., 2021 [29]
Fructose (1 g/kg fructose up to maximum of 25 g)	Hydrogen and methane	Rise of ≥ 20 ppm over baseline hydrogen; or ≥ 10 ppm over baseline methane. Test duration of 3 h	60% (by hydrogen), 35% (by methane) in pediatric patients with abdominal symptoms (n = 187)	Schneider et al., 2020 [30]
Fructose (40 g fructose in 330 cc water)	Hydrogen	Rise of ≥ 20 ppm over baseline hydrogen. Test duration of 3 h	65% in healthy patients (n = 20), 70% in IBS patients (n = 30)	Skoog et al., 2008 [31]
Fructose (1 g/kg fructose up to maximum of 50 g)	Hydrogen	Rise of ≥ 20 ppm over baseline hydrogen. Test duration of 3 h (symptoms monitored for 24 h)	68% in patients with abdominal pain (n = 31)	Ozaki et al., 2018 [32]
Fructose (A. 50 g fructose in 150 cc water, B. 50 g fructose in 250 cc water, C. 25 g in 250 cc water)	Hydrogen and methane	Rise of ≥ 3 ppm over 3 consecutive breath samples from baseline; or ≥ 20 ppm over baseline hydrogen and/or methane. Test duration of	74% (A, n = 203), 70% (B, n = 33), 39% (C, n = 36) in patients with abdominal symptoms	Choi et al., 2003 [33]
Fructose (1 g/kg fructose up to maximum of 50 g)	Hydrogen and methane	Rise of ≥ 20 ppm over baseline hydrogen and/or methane. Test duration of 5 h	59% in healthy pediatic patients (n = 34), 41% in pediatric patients with functional abdominal pain (n = 71)	Martínez-Azcona et al., 2019 [34]
Fructose (25 g fructose in 250 cc water)	Hydrogen	Rise of ≥ 20 ppm over baseline hydrogen. Test duration of 2 h	14% in healthy Thai patients (n = 77)	Densupsoontorn et al., 2007 [35]
Fructose (2 g/kg fructose up to maximum of 50 g)	Hydrogen	Rise of ≥ 20 ppm over baseline hydrogen. Test duration not reported	62% in pediatric patients with recurrent abdominal pain (n = 121)	Gijsbers et al., 2012 [36]
Fructose (25 g fructose in 250 cc water)	Hydrogen and methane	Hydrogen and/or methane levels ≥ 20 ppm. Test duration of 5 h	35% in IBS patients (n = 90)	Melchior et al., 2014 [37]
Fructose (A. 1 g fructose, B. 15 g fructose, C. 45 g fructose in 240 cc water)	Hydrogen	Rise of ≥ 20 ppm over baseline hydrogen. Test duration of 3 h	0% (A, n = 9), 3% (B, n = 10), 62% (C, n = 13) in pediatric patients with abdominal pain	Gomara et al., 2008 [38]
Fructose (50 g fructose in 250 cc water)	Hydrogen	Rise of ≥ 20 ppm over baseline hydrogen. Test duration of 3 h	75% in obese African American patients (n = 16), 43% in obese Hispanic patients (n = 21)	Walker et al., 2012 [39]
Fructose (25 g fructose in 200 cc water)	Hydrogen	Rise of ≥ 20 ppm over baseline hydrogen. Test duration of 2 h	24% in patients with abdominal symptoms (n = 341)	Enko et al., 2016 [40]
Fructan breath tests				
Fructan (10 g inulin dissolved in 100 cc water)	Hydrogen	Rise of ≥ 20 ppm over baseline hydrogen. Test duration of 3 h	19% in IBS patients (n = 21), 5% in healthy patients	Gutierrez et al., 2016 [41]
Fructan (10 g fructan dissolved in 100 cc water)	Hydrogen and methane	Rise of ≥ 20 ppm over baseline hydrogen; or ≥ 15 ppm over baseline methane. Test duration of 3 h	64% in IBS patients (n = 102)	Leelasinjaroen et al., 2017 [42]
Fructan (10 g fructan dissolved in 100 cc water)	Hydrogen and methane	Rise of ≥ 20 ppm over baseline hydrogen; or ≥ 15 ppm over baseline methane. Test duration of 3 h	58.3% in IBS patients (n = 24)	Yu et al., 2014 [43]
Fructan (10 g fructan dissolved in 100 cc water)	Hydrogen and methane	Rise of ≥ 20 ppm over baseline hydrogen; or ≥ 15 ppm over baseline methane. Test duration of 3 h	77% in female IBS patients	Attaluri et al., 2009 [44]

Previous work has shown that fructose- and fructan-free diets had high adherence and symptom improvement in patients with IBS and fructose malabsorption diagnosed by HBT [1]. Though fructose and fructan are structurally related carbohydrates, fructose and fructan malabsorption have never been described in the same patient cohort. Therefore, in this study, we investigate the possible association between fructose malabsorption and fructan malabsorption in patients with IBS.

## Methods

### Study cohort

A retrospective chart review was performed at a single medical clinic in the northeastern United States using records from January 2017 to June 2022. The electronic medical record was searched for patients with IBS (International Classification of Diseases, Tenth Revision code K58.0, K58.1, K58.2, K58.8, K58.9) who had undergone HBTs. Patients were excluded from our study for the following reasons: 1) The patient did not undergo a fructose HBT; 2) The patient did not undergo a fructan HBT; 3) SIBO had not been ruled out with a glucose HBT as previously described [1], or if the patient had a documented history of recurrent SIBO; and 4) The patient had previously been diagnosed with a secondary cause of malabsorption (celiac disease, inflammatory bowel disease, liver disease, pancreatic disease, and lymphatic disease) [19]. Though there is an association between SIBO and IBS [45], SIBO may cause false positives in fructose and fructan HBTs. Thus, patients with recurrent SIBO and patients who did not have SIBO ruled out prior to fructose and fructan HBT were excluded to eliminate false positives which may artificially inflate the association between fructose malabsorption and fructan malabsorption [18, 46]. This study was deemed to be exempt from institutional review board approval by WCG IRB. Therefore, the need for patient consent was waived.

### Fructose and fructan hydrogen breath testing

Patients were instructed to eat a low carbohydrate dinner the day before and to consume nothing by mouth for at least 12 h prior to their HBT. An initial baseline hydrogen reading was taken before the fructose or fructan solution was administered. If the baseline was determined to be < 20 ppm, the patient was said to have followed the preparatory instructions properly, as basal hydrogen levels were low enough to proceed.

A fructose solution (25 g fructose dissolved in 250 cc water; NOW Foods) or a fructan solution (10 g inulin dissolved in 250 cc water; Earthborn Elements) was administered to the patient to assess for fructose malabsorption or fructan malabsorption respectively, hydrogen levels were noted every 30 min for the next 3 h. Breath samples were assayed for hydrogen levels using a Gastrolyzer/Gastro^+^ ™ (Bedfont® Scientific Ltd, UK). The same device was used for all patients, and it was recalibrated every month to ensure accurate hydrogen readings. Patients were considered positive for fructose malabsorption or fructan malabsorption if hydrogen levels were ≥ 20 ppm [21, 37]. If a patient was determined to be positive for fructose malabsorption or fructan malabsorption before 3 h had elapsed, the test administrator ended the data collection period early. Throughout the study, we use the term “malabsorption” determined by hydrogen gas readings, as opposed to “intolerance”, which would necessitate symptom quantification during the test period.

After patients underwent fructose or fructan HBT, they returned to the clinic on a separate day (minimum 1 day between HBTs) having followed the same preparation. Neither the patient nor the HBT administrator were blinded to the test substance.

### Data abstraction and analysis

Patient demographics, clinical characteristics, and HBT results were abstracted from the electronic medical record. Data was independently abstracted by at least two abstractors. A third abstractor resolved any conflicting data. During data abstraction, all abstractors were blinded to the study hypotheses.

Descriptive statistics were used to analyze patient demographics and clinical characteristics. Using the Shapiro–Wilk test, our hydrogen values were not found to have a normal distribution. Therefore, hydrogen levels are reported as medians and quartiles. Differences in median hydrogen levels between the patients who tested positive and negative were calculated with the Mann–Whitney test. A two-sided chi-squared test was used to compare fructose HBT results in patients positive for fructan malabsorption versus patients who were negative for fructan malabsorption, and vice versa. Odds ratios were reported for significant results. Statistical analysis was performed in GraphPad Prism 10 (GraphPad Software Inc.; San Diego, CA, USA).

## Results

### Patient cohort

**Fig. 1:**
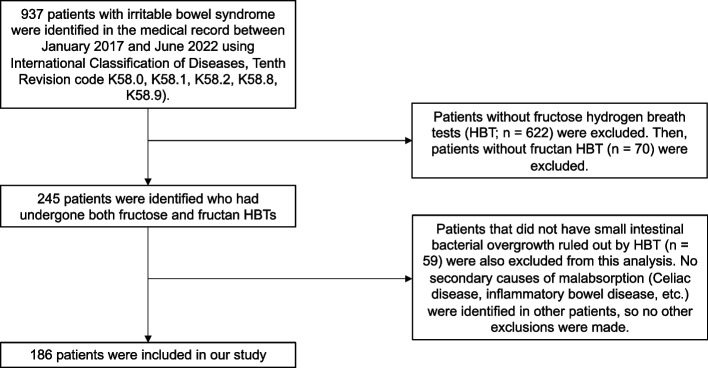
Flowchart of included and excluded patients in our retrospective chart review

Out of 937 patients identified with IBS, 245 were identified in the electronic medical record to have undergone both fructose and fructan HBTs between January 2017 and June 2022. Of these patients, 59 were excluded due to being unable to rule out underlying SIBO. None of the remaining patients were diagnosed with secondary causes of malabsorption. Therefore, 186 patients were included in our retrospective study (Fig. 1).

The patient cohort had a median age of 36.7 (IQR 29.6–47.5). Seventy patients (37.6%) were male. Patients presented with various symptoms, with the most reported gastrointestinal complaints including bloating (133 patients, 71.5%), abdominal pain (118 patients, 63.4%), and diarrhea (79 patients, 42.5%; Table 2).

**Table 2:** Demographics and clinical presentation of patients who had fructose or fructan hydrogen breath tests (HBTs)

Characteristics	All Patients (*n* = 186)	Patients Positive for Fructose HBT (*n* = 71)	Patients Negative for Fructose HBT (*n* = 115)	Patients Positive for Fructan HBT (*n* = 91)	Patients Negative for Fructan HBT (*n* = 95)	Patients Positive for Both HBTs (*n* = 42)	Patients Negative for Both HBTs (*n* = 66)
Age, median years (Q1-Q3)	36.7 (29.6–47.5)	38.8 (29.8–51.2)	34.8 (28.5–43.6)	37.6 (29.8–47.2)	34.8 (28.6–46.4)	39.9 (29.8–52.3)	34.0 (28.0–43.5)
Male, n (%)	70 (37.6)	26 (36.6)	43 (37.4%)	30 (33.0%)	41 (43.2%)	17 (40.5%)	29 (43.9%)
Symptoms, n (%)							
Bloating	133 (71.5%)	47 (66.2%)	86 (74.8%)	68 (74.7%)	65 (68.4%)	27 (67.3%)	45 (68.2%)
Abdominal Pain	118 (63.4%)	40 (56.3%)	78 (67.8%)	58 (63.7%)	60 (63.2%)	23 (54.8%)	43 (65.2%)
Diarrhea	79 (42.5%)	30 (42.3%)	49 (42.6%)	39 (42.9%)	40 (42.1%)	19 (45.2%)	29 (43.9%)
Constipation	48 (25.8%)	19 (26.8%)	29 (25.2%)	27 (29.7%)	21 (22.1%)	13 (31.0%)	15 (22.7%)
Increased Flatulence	28 (15.1%)	11 (15.5%)	17 (14.8%)	11 (12.1%)	17 (17.9%)	5 (11.9%)	11 (16.7%)
Nausea	29 (15.6%)	11 (15.5%)	18 (15.7%)	19 (20.9%)	10 (10.5%)	7 (16.7%)	6 (9.1%)
Acid Reflux	23 (12.4%)	5 (7.0%)	18 (15.7%)	10 (11.0%)	13 (13.7%)	0 (0.00%)	8 (12.1%)
Fecal Urgency	25 (13.4%)	9 (12.7%)	16 (13.9%)	14 (15.4%)	11 (11.6%)	7 (16.7%)	9 (13.6%)
Weight Loss	12 (6.5%)	4 (5.6%)	8 (7.0%)	6 (6.6%)	6 (6.3%)	1 (2.4%)	3 (4.6%)
Vomiting	8 (4.3%)	4 (5.6%)	4 (3.5%)	5 (5.5%)	3 (3.2%)	3 (7.1%)	2 (3.0%)
Appetite Loss	9 (4.8%)	4 (5.6%)	5 (4.4%)	3 (3.3%)	6 (6.3%)	1 (2.4%)	3 (4.6%)
Nonspecific Extraintestinal Complaints	36 (19.4%)	12 (16.9%)	25 (20.9%)	13 (14.3%)	23 (24.2%)	5 (11.9%)	16 (24.2%)

Because there may be a potential selection bias that only patients with reported fructose and fructan-associated triggers would undergo both fructose and fructan HBT, we assessed the proportion of patients with reported triggers of any food, fructose-containing food, or fructan-containing food. Of 751 patients excluded from our analysis, 567 (75.5%) had food-related complaints, 80 (10.7%) reported issues with fructose-containing foods, and 117 (15.6%) had issues with fructan-containing foods. Of the 186 patients included in our study, 154 (82.8%) had food-related complaints, 16 (8.6%) reported issues with fructose-containing foods, and 33 (17.7%) had issues with fructan-containing foods. Using two-sided chi-squared tests, we compared the proportion of reported triggers of any food, fructose-containing foods, and fructan-containing foods between patients who were excluded versus patients who were included in this study cohort. There were significantly increased reports of food triggers in included patients (82.8%) versus excluded patients (75.5%; *p* = 0.0344). In contrast, there was no difference in reported triggers of fructose-containing foods in included patients (8.6%) versus excluded patients (10.7%; *p* = 0.4901). Similarly, there was no difference in reported triggers of fructan-containing foods in included patients (17.7%) versus excluded patients (15.6%; *p* = 0.4714). Therefore, although patients with food-related complaints were more likely to be included in the study, the specific foods were not biased toward fructose- or fructan-containing foods.

### Fructose and fructan hydrogen breath tests

All 186 patients in the cohort underwent both fructose and fructan HBTs, with 71 patients (38.2%) testing positive for fructose malabsorption, and 91 patients (48.9%) testing positive for fructan malabsorption. Of these, 42 patients (22.6%) were positive for both fructose malabsorption and fructan malabsorption (Table 2, Supplementary Fig. 1, Supplementary Table 1). Hydrogen readings for all fructose and fructan HBTs are summarized in Supplementary Fig. 1 and Supplementary Table 1. The average time for positive readings was 57.0 min (SD 23.7) for fructose HBTs, and 110.4 min (SD 46.6) for fructan HBTs (Supplementary Table 1).

Average washout period between HBTs was 12 days (SD 10, minimum = 1 day, maximum = 42 days). To investigate if there were sequence effects, 98 patients (52.7%) underwent fructose HBT first, and 88 patients (47.3%) underwent fructan HBT first. Test results were compared in patients who underwent fructose HBT versus fructan HBT first using a two-sided chi-square test. The proportion of patients positive for fructose malabsorption who did fructose HBT first (*n* = 38/98; 38.8%) was not significantly different from those who did fructan HBT first (*n* = 33/88; 37.5%; *p* = 0.8581). Similarly, the proportion of patients positive for fructan malabsorption who did fructose HBT first (*n* = 45/98; 45.9%) was not significantly different from those who did fructan HBT first (*n* = 46/88; 52.3%; *p* = 0.3867).

### Relationship of fructose malabsorption and fructan malabsorption

**Fig. 2:**
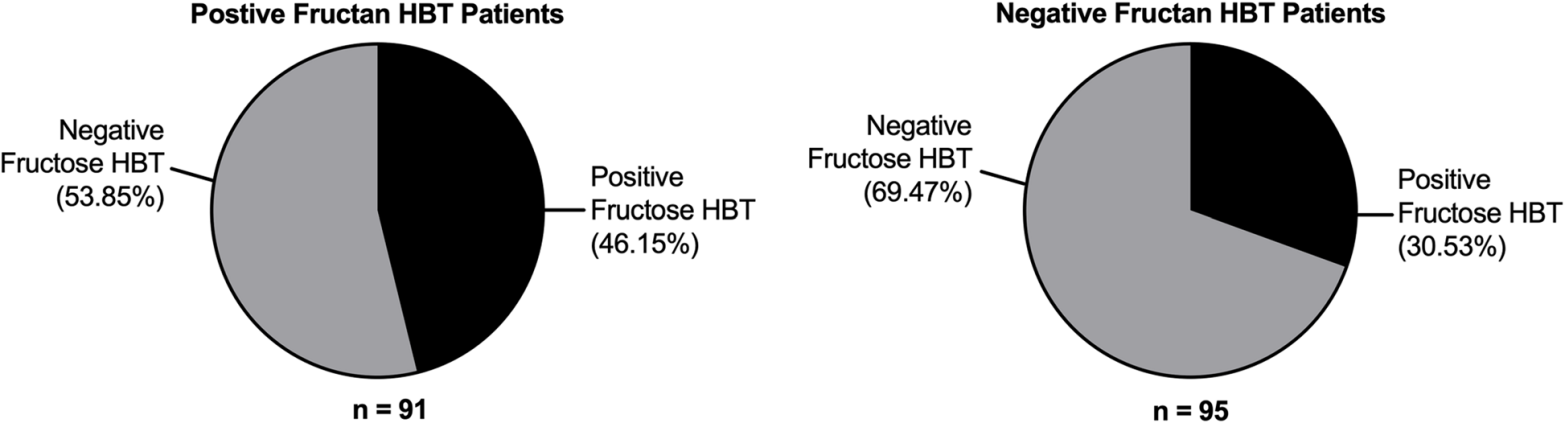
Hydrogen breath test results for fructose and fructan malabsorption testing (*n* = 186). Fructose HBT test results are separated based on fructan HBT result (positive, *n* = 91; negative, *n* = 95). Results for fructose and fructan HBTs were compared using a two-sided chi-squared test to evaluate correlation between HBT results for fructose and fructan (*p* = 0.0283, OR 1.951, 95% CI 1.072–3.476)

To investigate if there was any correlation between fructose and fructan HBT results, we compared fructose HBT results in patients positive for fructan malabsorption versus patients who were negative for fructan malabsorption, and vice versa. We found that there was a significant difference in the proportion of positive fructose or fructan HBTs between patients who tested positive and patients who tested negative for the other HBT (*p* = 0.0283; Fig. 2). Patients who tested positive for fructose malabsorption or fructan malabsorption had 1.951 times (95% CI 1.072–3.476) higher odds of testing positive for the other carbohydrate.

## Discussion

Though fructose and fructan are closely related FODMAPs that have been suggested to be possible triggers of IBS, fructose malabsorption and fructan malabsorption have never been studied in the same patient population. In the present study, we show that out of 186 patients with IBS who were tested for both fructose and fructan malabsorption by HBT, 71 patients (38.2%) were positive for fructose malabsorption, and 91 patients (48.9%) were positive for fructan malabsorption. Of these positive results, 42 patients (22.6%) were positive for both fructose malabsorption and fructan malabsorption. Crucially, we also found that patients who were positive for fructose malabsorption or fructan malabsorption had 1.951 times higher odds of testing positive for the other carbohydrate. Previous work showed that fructose- and fructan-free diets improved symptoms in patients with IBS and fructose malabsorption [36–39]. Our results support their findings, as almost a quarter (22.6%) of tested patients were positive for both fructose and fructan malabsorption. However, our findings also show that 41.9% of patients who were positive for one carbohydrate but not the other, illustrating the potential drawback of empirically eliminating both fructose and fructans in patients who have one malabsorption. In other words, though elimination of both carbohydrates may improve potential clinical benefit in some patients (22.6%), it may be needlessly restrictive in a larger proportion of patients (41.9%). Instead, the positive association between fructose and fructan malabsorption in patients with IBS suggests that fructan malabsorption should be suspected in a patient who tests positive for fructose malabsorption, and vice versa.

Further research is needed to understand the proximate mechanisms underlying the positive association between fructose and fructan malabsorption. In several studies, increased fructose and fructans in the gastrointestinal tract have been shown to profoundly alter the gut microbiome [6, 47–53]. Therefore, patients with malabsorption to one carbohydrate may result in dysbiosis of the gut, and thus, malabsorption of the other carbohydrate as well. For example, diets that increase fructose in the gastrointestinal tract have been shown to shift intestinal populations of bacteria containing fructan hydrolases (Actinobacteria and Firmicutes) [54], thus promoting fructan fermentation. Alternatively, previous studies have hypothesized that fructose malabsorption is a result of disrupted fructose transporters, while in fructan malabsorption, a lack of hydrolytic enzymes results in a hypersensitivity response in patients with IBS [3, 17]. Another possible explanation for our findings is that fructan may be spontaneously breaking down into fructose in small amounts in the gastrointestinal tract, which may overwhelm defective fructose transporter proteins. Thus, in this scenario, a patient with fructose malabsorption may also exhibit malabsorption when ingesting fructans.

Our findings also provoke additional research questions. Previous work by Wilder-Smith et al. has found that lactose and fructose malabsorption co-occur in 16% of patients with gastrointestinal disease [24]. Future work should investigate the possible co-occurrence of lactose, fructose, and fructan malabsorption. If the three co-occur together, this would support a neurologic or microbiomic mechanism, as opposed to GLUT5-dependent mechanism.

There are limitations associated with our HBT protocol. In this study, we only present hydrogen gas data, though methane breath tests have also been previously investigated in the context of carbohydrate malabsorption. Our criteria for positive fructose and fructan malabsorption used an absolute hydrogen threshold of 20 ppm. Though this criterion has been used in several studies [21, 37], it differs from the majority of current literature (Table 1). Our maximum HBT duration was 3 h, which is consistent with previous literature on fructan HBT and most of the previous literature on fructose HBT (Table 1). However, rises in hydrogen gas may have occurred after 3 h. Thus, we may have underestimated the proportion of patients with fructose and fructan malabsorption. Despite this, our finding of the association of fructose and fructan malabsorption in patients with IBS remains supported given that we used a uniform testing protocol for all patients included in this study. The specific fructan chain length used in this study is unknown, as it is not reported by the manufacturer. Fructan chain length has never been reported in any previous work on fructan HBT. Limited evidence has suggested that breath hydrogen is higher when participants consume longer-chain fructan-containing food versus shorter-chain fructan-containing food, thus possibly affecting our results [55]. Future work on fructan HBT should investigate the role of fructan chain length in HBT and if it influences the sensitivity and specificity for fructan intolerance.

Other limitations of our study include the retrospective study design. We are limited by the completeness of the electronic medical record which precludes certain kinds of analyses such as quantification of symptoms during HBT. However, previous work has shown that symptoms and gas readings during HBTs are correlated [26]. Our study is a single-site study with patients primarily from the northeastern region of the United States. We also lacked data on race/ethnicity. Thus, there may be limited generalizability of our findings. Furthermore, our research does not address if the association of fructose malabsorption and fructan malabsorption is a feature unique to patients with IBS, or if this association is also present in a normal, healthy population or other functional gastrointestinal disorders, such as functional dyspepsia. Further research should seek to address these limitations with prospective, case-controlled clinical trials.

## Conclusion

Our study is the first to investigate the association between fructose malabsorption and fructan malabsorption in the same patients with IBS. Through retrospective analysis of IBS patients who underwent both fructose and fructan HBT, patients with either a positive fructan or fructose HBT had higher odds of testing positive for the other carbohydrate. Therefore, fructan malabsorption should be suspected in a patient with fructose malabsorption, and vice versa.

### Supplementary Information

**Supplementary Material 1.**

## Data Availability

All relevant de-identified data and study materials are stored in a HIPPA-compliant, password protected, cloud-based storage. Access to these files will be provided upon reasonable request to the corresponding author.

## Abbreviations

HBT: Hydrogen breath test
IBS: Irritable bowel syndrome
SIBO: Small intestinal bacteria overgrowth
