# Supplementary material for: Fructose malabsorption and fructan malabsorption are associated in patients with irritable bowel syndrome
Source: BMC Gastroenterol. 2024 Apr 24;24:143. doi: 10.1186/s12876-024-03230-x (PMC11040878; doi:10.1186/s12876-024-03230-x)
Supplement: Supplementary file 1 — Supplementary Material 1. [file 12876_2024_3230_MOESM1_ESM.docx]

**SUPPLEMENTARY MATERIALS**

**Fructose malabsorption and fructan malabsorption are associated in patients with irritable bowel syndrome**

Twan Sia^1, 2^, Riki O. Tanaka^1^, Albert Mousad^3^, Aditya Narayan^2^, Kristen Si^1^, Leeon Bacchus^1^, Hind Ouerghi^1^, Aashka Patel^1^, Arnav Patel^1^, Evan Cunningham^1^, Taylor Epstein^1^, Jerry Fu^1^, Stanley Liu^1^, Raisa Khuda^1^, Paige McDonald^1^, Shibani Malik^1^, Joanna McNulty^1^, Michelle Pan^1^, John Leung^1^*

^1^Boston Specialists

65 Harrison Ave #201

Boston, Massachusetts 02111

^2^Stanford University School of Medicine

291 Campus Drive

Stanford, CA 94305

^3^Tufts University School of Medicine

145 Harrison Ave

Boston, MA 02111

*Corresponding Author

John Leung, MD

Boston Specialists

65 Harrison Ave #201

Boston, Massachusetts 02111

[drjohnleung@bfac.org](mailto:drjohnleung@bfac.org)

**This file includes:** Figure S1, Table S1.

**Supplementary Table 1. Hydrogen levels for fructose and fructan hydrogen breath tests (HBTs)**

| Results | Patients Positive for Fructose HBT (n=71) | Patients Negative for Fructose HBT (n=115) | Patients Positive for Fructan HBT (n=91) | Patients Negative for Fructan HBT (n=95) |
| --- | --- | --- | --- | --- |
| H2 Readings, median ppm (Q1-Q3), n |  |  |  |  |
| Baseline | 5.0 (3.0-10.0), 71 | 3.0 (2.0-5.0), 115 | 4.0 (2.0-7.0), 91 | 3.0 (2.0-6.0), 95 |
| 30 Mins After Fructose/Fructan Administration | 13.0 (8.0-22.0), 71 | 4.0 (3.0-7.0), 115 | 6.0 (3.0-10.0), 91 | 4.0 (3.0-6.0), 95 |
| 60 Mins After Fructose/Fructan Administration | 27.0 (18.0-37.0), 49 | 5.0 (3.0-7.5), 115 | 7.0 (3.0-14.5), 81 | 3.0 (2.0-6.0), 95 |
| 90 Mins After Fructose/Fructan Administration | 28.5 (19.0-36.0), 14 | 4.0 (3.0-7.0), 115 | 9.0 (4.5-22.5), 69 | 3.0 (2.0-5.0), 95 |
| 120 Mins After Fructose/Fructan Administration | 23.5 (16.8-43.8), 4 | 4.0 (2.0-6.0), 115 | 16.5 (8.0-25.5), 50 | 3.0 (3.0-5.5), 95 |
| 150 Mins After Fructose/Fructan Administration | 16.0 (16.0-16.0), 1 | 3.0 (2.0-5.0), 113 | 20.5 (12.3-26.0), 30 | 4.0 (3.0-7.0), 92 |
| 180 Mins After Fructose/Fructan Administration | N/A | 3.0 (2.0-5.0), 92 | 27.0 (22.0-31.0), 14 | 6.0 (3.0-10.0), 80 |
| Average Time to H2 >= 20 ppm, Mean Minutes +/- SD | 57.0 +/- 23.7 | 3.0 +/- 0.0 | 110.4 +/- 46.6 | N/A |


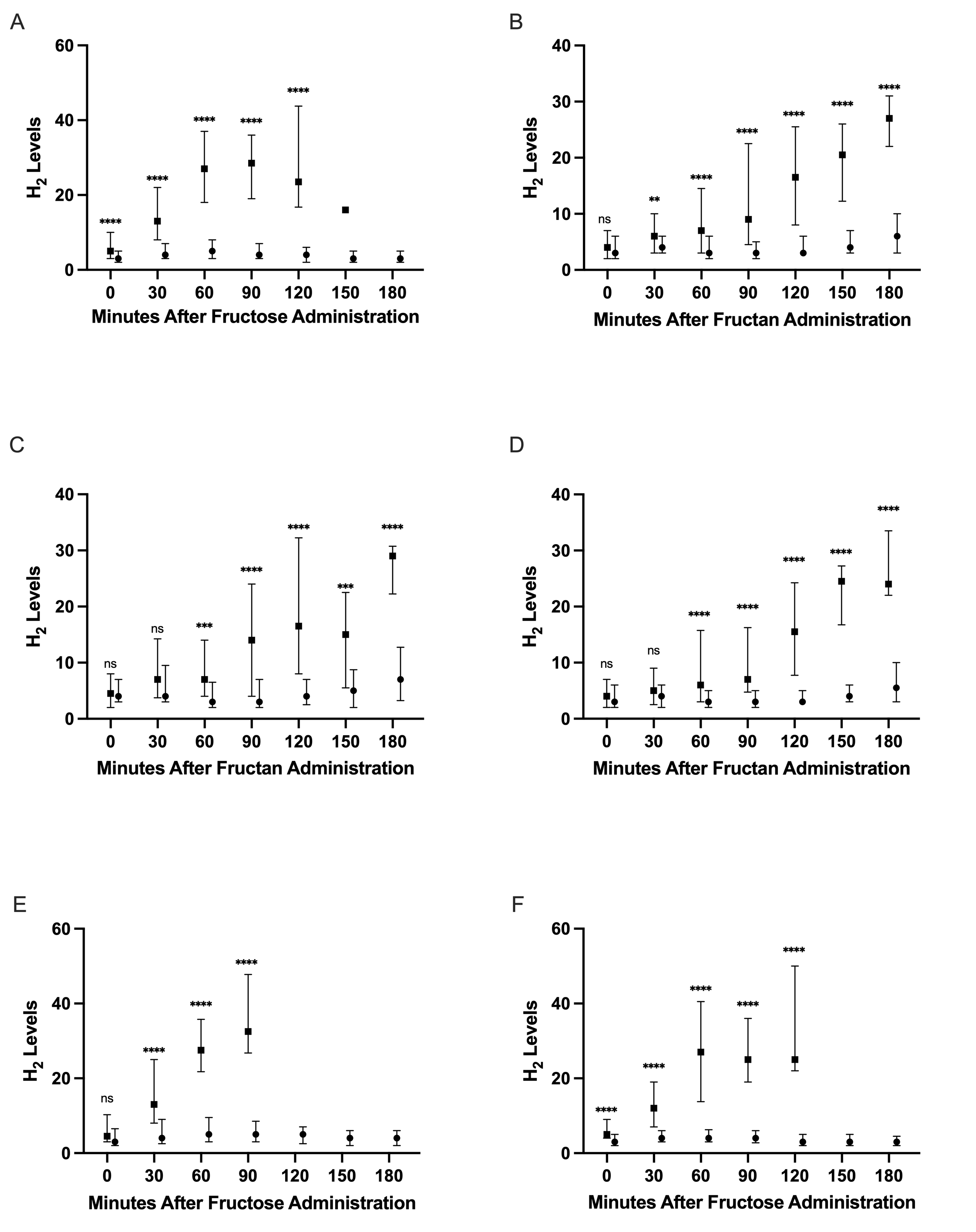


**Supplementary Figure 1.** Hydrogen levels for fructose and fructan HBTs (n = 186). Results for fructan HBTs were analyzed based on whether the patient has tested positive or negative for fructose malabsorption and vice versa. Unpaired Mann Whitney U test was used to compare median hydrogen levels for positive and negative HBTs for each subpopulation. (**A**) All patients that took fructose HBTs were separated into positive and negative fructose HBT groups. Hydrogen levels were compared at baseline (p = 0.00006), 30 (p <0.000001), 60 (p <0.000001), 90 (p <0.000001), and 120 (p = 0.000002) minutes. There was an insufficient number of positive tests at 150 and 180 minutes for comparison. (**B**) All patients that took fructan HBTs were separated into positive and negative fructan HBT groups. Hydrogen levels were compared at baseline (p = 0.23562), 30 (p = 0.00773), 60 (p < 0.000001), 90 (p < 0.000001), 120 (p < 0.000001), 150 (p < 0.000001), and 180 minutes (p < 0.000001). (**C**) Patients that tested positive for fructose malabsorption that took fructan HBTs were separated into positive and negative fructan HBT groups. Hydrogen levels were compared at baseline (p = 0.69285), 30 (p = 0.05307), 60 (p = 0.006663), 90 (p = 0.000129), 120 (p < 0.000023), 150 (p = 0.002114), and 180 minutes (p < 0.000001). (**D**) Patients that tested negative for fructose malabsorption that took fructan HBTs were separated into positive and negative fructan HBT groups. Hydrogen levels were compared at baseline (p = 0.19559), 30 (p = 0.146982), 60 (p = 0.000052), 90 (p < 0.000001), 120 (p < 0.000001), 150 (p < 0.000001), and 180 minutes (p < 0.000001). (**E**) Patients that tested positive for fructan malabsorption that took fructose HBTs were separated into positive and negative fructose HBT groups. Hydrogen levels were compared at baseline (p = 0.099698), 30 (p < 0.000001), 60 (p < 0.000001), and 90 (p < 0.000001) minutes.  There was an insufficient number of positive tests at 120, 150, and 180 minutes for comparison. (**F**) Patients that tested negative for fructan malabsorption that took fructose HBTs were separated into positive and negative fructose HBT groups. Hydrogen levels were compared at baseline (p = 0.000044), 30 (p < 0.000001), 60 (p < 0.000001), 90 (p < 0.000001), and 120 (p = 0.000019) minutes. There was an insufficient number of positive tests at 150 and 180 minutes for comparison. Squares represent medians for positive HBT hydrogen readings. Circles represent medians for negative HBT hydrogen readings. Error bars represent IQR. Asterisks denote significant differences (**** p < 0.0001, *** p < 0.001, ** p < 0.01, * p < 0.05).
